# Cement-Treated Volcanic Scoria for Low-Traffic Road Pavements in the Azores Archipelago

**DOI:** 10.3390/ma14206080

**Published:** 2021-10-14

**Authors:** João Crucho, Luís Picado-Santos, Filipe Silva

**Affiliations:** 1 CERIS, Instituto Superior Técnico, Universidade de Lisboa, Av. Rovisco Pais, 1049-001 Lisboa, Portugal; 2 Instituto Superior Técnico, Universidade de Lisboa, Av. Rovisco Pais, 1049-001 Lisboa, Portugal; silva.filipegomes@gmail.com

**Keywords:** volcanic scoria, basaltic pyroclasts, cement-treated materials, low-traffic road pavements

## Abstract

The Azoreans rely on an extensive network of rural roads for the most of the rural population’s activities (primary sector) and accessibility. To rehabilitate and maintain this network, asphalt concrete and crushed rock aggregate are usually used. However, in the region, the application of such paving technology can be extremely costly. It requires specialized contractors, dedicated equipment and raw materials that must be imported to most of the islands. Therefore, the use of locally available materials would result in more flexibility and fewer costs for planned interventions. In the Azores, known as *bagacina*, the volcanic scoria is a pyroclastic material, generally highly abundant in volcanic islands. This natural aggregate is inexpensive, easy to extract, and presents good geotechnical characteristics. However, due to its porous nature, it generally does not comply with the current specifications for pavement materials. Therefore, this study aims to evaluate cement-treated volcanic scoria to be used as low-traffic road pavement layers. The geotechnical properties and mechanical performance of the two types of scoriae were analyzed. As a result, both types of scoriae presented good behavior, according to the expected for a cement-treated material, and proved to be a suitable alternative for road pavements in the Azores Archipelago.

## 1. Introduction

The archipelago of the Azores, an autonomous Portuguese region, is a part of Macaronesia, a group of volcanic islands in the North Atlantic Ocean. In the Azores, the primary sector, which is mainly agriculture and livestock, represents substantial economic activity [1]. The region has an extensive network of agricultural and rural roads essential for the local economy and crucial accessibility for most of the rural population. Over the past decade, a significant economic effort has been made by local authorities to rehabilitate and improve many of these roads, usually using asphalt concrete and crushed rock aggregate. However, in the region, the application of such paving technology can be extremely costly, as it requires specialized private contractors, specific equipment and raw materials that have to be imported. For the transport of such cargo, the only option is through shipping. As the distance between the Azores and Portuguese continental land is approximately 1500 km, and within the archipelago, the distance between the most distant islands is over 600 km, the transport is complex, costly and time-consuming. In addition, particularly in winter, the sea and weather conditions can disrupt transport. This solution represented a high inefficiency for the equipment because of the time consumed during transport and increased the risk of failure/malfunction due to eventual damage associated with the transport. 

The use of more simple equipment and locally available materials could promote a more economic and environmentally friendly solution. Besides the environmental gains, eliminating the constraints associated with transport will allow more flexibility in planning interventions. In addition, a reduction in the unitary costs will enable a higher intervention area, thus, an overall increase in the rural network quality. 

In volcanic islands, volcanic scoria is an abundant material. The fragmentation of magma and rock forms these materials through explosive volcanic activity, common in Strombolian-type eruptions [2]. Regarding geochemical composition, the pyroclastic materials can be more basaltic or pumice. The pumice deposits are designated as pumice, and the basaltic deposits are designated as scoria. Thus, the volcanic scoria is loose or weakly cemented basaltic pyroclasts that settle near the eruptive center [3]. They are dark-colored, typically dark-gray to black or red/purplish-red, dependent on whether the iron has oxidized. Volcanic scoria can be considered a natural aggregate, inexpensive, easy to extract, and with good geotechnical characteristics. Generally, these materials present a well-graded particle size distribution, non-plasticity, good drainage properties and acceptable bearing capacity [4]. Regarding bearing capacity, CBR from 10% to 60% can be expected [5,6]. However, due to the particle’s high void content, they tend to be vulnerable to impact, abrasion and fragmentation [7,8]. 

The Portuguese technical specifications were established according to the technology and knowledge derived from the Iberian Peninsula materials. These materials exhibit different lithology (for instance, limestone and granite aggregates). Thus, it can be difficult for the volcanic scoria, and volcanic materials in general, to fully comply with those specifications. For instance, in the Canary Islands (Spanish archipelago situated southeast of Azores, near the African coast), the scoria is mainly used to form subgrades. The reason is that they tend to not complying with the Spanish road standard specifications [9] for fracture strength, gradation and sand equivalent [10]. Traditionally, volcanic scoria has been used as unbound granular layers, such as, base, subbase, subgrade or even the surface on rural low-traffic roads [4]. More recently, they are often applied as aggregate for insulation, lean concrete, cement mortars, and pre-cast concrete elements such as bricks and joists. In addition, some studies already validate the use of these types of aggregates in lightweight concrete applications [11,12,13,14,15]. The alkaline composition of volcanic scoria, with low silica content, eliminates alkali–silica reactivity with Portland cement [2,12] and makes it a material suitable for cement-bound granular layers.

Generally, for low-traffic road pavements, no wearing course is laid to protect the unbound scoria granular layer, i.e., the granular layer of scoria is in direct contact with traffic. Therefore, pavement’s durability is lower due to the disaggregation and loss of material on the trafficked surface. The use of cement on those layers, a commonly available binder easy to stock, would bring additional durability and bearing strength to the pavement.

This study evaluates the technical feasibility of cement-treated volcanic scoria (CTS) as a cost-effective alternative to the current pavement technology being applied for low-traffic roads in the Azores Archipelago. For this evaluation, two types of scoriae, extracted from different scoria cones, were studied. Firstly, their geotechnical properties were characterized, and later, their mechanical performance was evaluated through unconfined compressive strength (UCS) and indirect tensile strength (ITS) tests.

## 2. Materials and Methods

### 2.1. Materials and Sample Preparation

**Figure 1:**
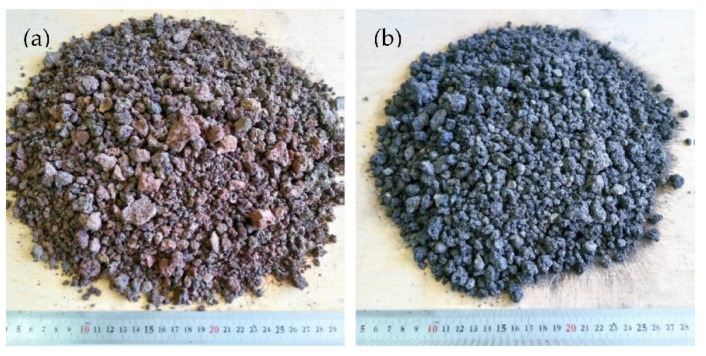
Sample of red scoria (**a**) and black scoria (**b**).

To evaluate the suitability of the cement-treated scoria (CTS) two types of volcanic scoria were tested. Visually, the scoria types differ in color. One type is red/purplish-red color (further designated as red), and the other type is dark-grey (further designated as black). Figure 1 presents a sample of both scoria types. The scoriae were obtained from two different scoria cones on the island of *São Jorge* in the Azores archipelago. 

The selected binder was the cement CEM II/B-L 32.5 N, a blended limestone Portland cement with a 28-day characteristic compressive strength of 32.5 MPa. This cement is readily available in the Portuguese market and can be supplied in bags, for instance, of 25 kg and 40 kg. This fact can facilitate transport to remote areas and enable small-scale applications without heavy specialized equipment.

**Figure 2:**
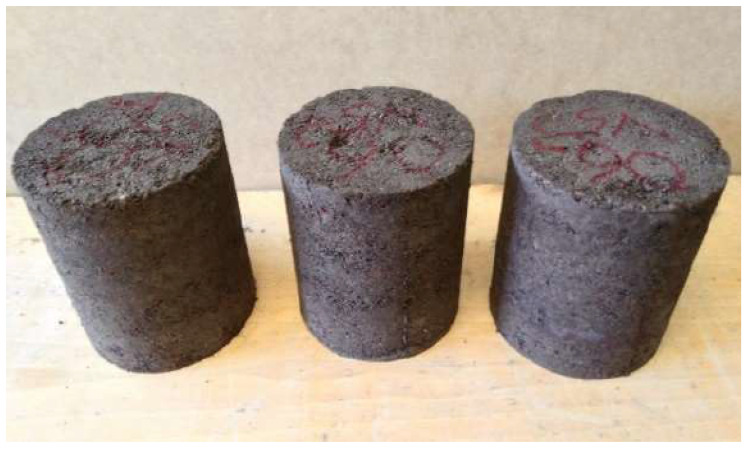
Example of three compacted specimens of CTS.

Following the geotechnical characterization of the materials, cylindrical specimens were molded (as presented in Figure 2) to evaluate the CTS’s compaction properties and mechanical performance. For the compaction properties, the cylindrical specimens were produced following the modified Proctor method, using the Proctor mold B (with 150 mm diameter and 120 mm height), compacted in five layers, applying 56 blows per layer with a 4.5 kg rammer, according to EN 13286-50 [16]. The cylindrical specimens for the mechanical performance tests were compacted following a similar procedure but using molds with a slenderness ratio of 1.15 (102 mm diameter and 117 mm height).

### 2.2. Test Methods

The geotechnical properties of both scoria types were characterized according to the test methods presented in Table 1. The maximum dry density and respective water content were determined by the modified Proctor compaction test, conducted according to EN 13286-2 [17]. Following the compaction tests, a new particle size distribution analysis was conducted and determined the respective fineness modulus. This analysis aimed to quantify the effects of compaction on the particle size distribution of the scoria materials.

The mechanical performance of the CTS was evaluated through unconfined compressive strength (UCS) and indirect tensile strength (ITS) according to EN 13286-41 [24] and EN 13286-42 [25], respectively. In both cases, strength was determined as the average of three individual results, at the curing ages of 7 days and 28 days. For curing, specimens were conditioned in the curing room at 20 ± 2 °C and relative humidity ≥ 95%, as indicated in EN 12390-2 [26]. The strength tests were conducted under constant deformation at 1.27 mm/min. For the UCS and ITS tests, the CTS specimens were compacted at the target water content determined in the respective compaction test. For the ITS tests, CTS specimens were produced with 4%, 5% and 6% of cement dosage (by dry mass), and for UCS, CTS specimens were made with 5% cement dosage. 

## 3. Results and Discussion

### 3.1. Geotechnical Properties

**Figure 3:**
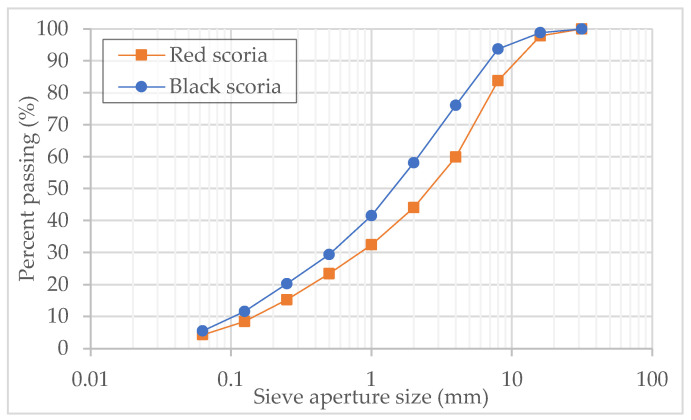
Particle size distribution of the red and black scoria.

The particle size distribution of both scoria types is presented in Figure 3. No particles were retained in the 31.5 mm sieve for both materials, and the quantity of fines (passing the 0.063 mm sieve) was approximately 5%. According to the Unified Soil Classification System (USCS), both materials qualified as well-graded sand. Compared to the black scoria, the red scoria showed a less uniform grading with a greater proportion of coarse particles.

Regarding the particle density and water absorption of the particles, a significant difference was identified between the finer (fraction 0.063/4) and coarser (fraction 4/16) particles (Table 2). The red scoria and black scoria presented a similar trend, with the fraction 0.063/4 exhibiting higher particle density and lower water absorption than the fraction 4/16. However, the differences were higher in the case of the black scoria. Compared to the red scoria, the black scoria presented higher particle density and lower water absorption in the fraction 0.063/4, and lower particle density and higher water absorption in the fraction 4/16. These results highlight the higher porosity of the coarser particles, and that black scoria is more sensitive than red scoria. Generally, traditional aggregates (e.g., limestone and granite) present higher particle density and lower water absorption. Therefore, the differences between conventional aggregates and scoria are particularly higher in the coarser fractions. For instance, limestone can present values of 2.66 Mg/m^3^ and 2.3% [27], granite, 2.68 Mg/m^3^ and 1.1% [28], and basalt, 2.95 Mg/m^3^ and 1.0% [29], for apparent particle density and water absorption, respectively.

The assessment of fines was conducted through the sand equivalent test and methylene blue test (Table 2). For both scoria types, the result of the sand equivalent test can be considered high (> 80%), greatly exceeding the typical threshold set by the infrastructure agencies. For instance, the Portuguese specification [30] requires a sand equivalent value ≥40%. On the one hand, both scoria types presented a similar proportion of fine particles regarding the methylene blue test result. On the other hand, the higher methylene blue value found for the red scoria is probably due to very plastic soil particles or organic matter present.

The content of organic matter was evaluated by igniting the oven-dried scoria in a furnace. Compared to the black scoria, the red scoria presented a higher content of organic matter (Table 2). However, for both scoriae, the content of organic matter is minimal, even negligible for the black scoria. Thus, problems in performance are not expected due to the organic matter content.

**Figure 4:**
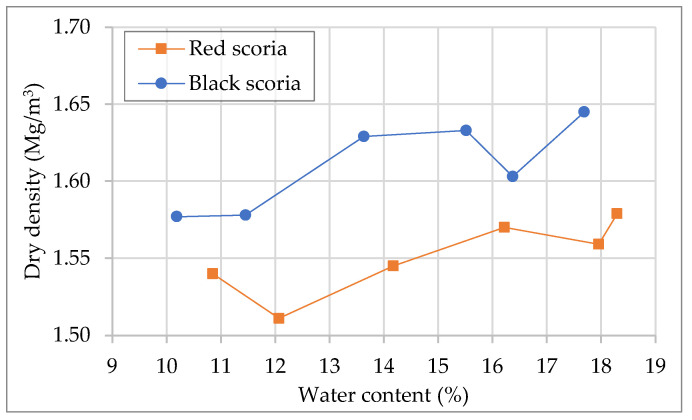
Dry density/water content relationship curves of the red and black scoria.

The compaction test results are expressed by the relation between dry density and water content (Figure 4). The maximum dry density values obtained were 1.58 Mg/m^3^ and 1.65 Mg/m^3^ for the red scoria and the black scoria. Both values of maximum dry density were obtained at approximately 18% water content. However, concerning water content, the variation in relative compaction was relatively low (under 5%). These results are in good agreement with the mentioned by Franesqui et al. [5], that for modified Proctor, indicates for this type of material, maximum dry density in the range from 0.8 Mg/m^3^ to 1.98 Mg/m^3^, and optimum moisture content in the range from 9% to 20%. Compared with results for other natural aggregates, scoria presents a poor relationship between water content and dry density (Figure 4), making it difficult to identify an obvious peak value for the dry density. At a higher water content, water leaked out from the base of the molds, resulting in a final water content lower than the target value. For instance, for the black scoria, the target water content of 20% and 18% resulted in actual water content of 18% and 16%, respectively. Thus, it is recommended to adopt optimum values within the range where the target content closely matches the actual content. For the characterization of mechanical performance, the specimens were produced with target water content of 14% and 12% for the red scoria and the black scoria, respectively. As expected, adding cement slightly increased the dry density of both materials. In the case of the red CTS, the dry density increased to 1.66 Mg/m^3^ and 1.68 Mg/m^3^ for the cement dosages of 4% and 6%, respectively. In the case of the black CTS, the dry density increased to 1.76 Mg/m^3^ and 1.77 Mg/m^3^ for the cement dosages of 4% and 6%, respectively.

**Figure 5:**
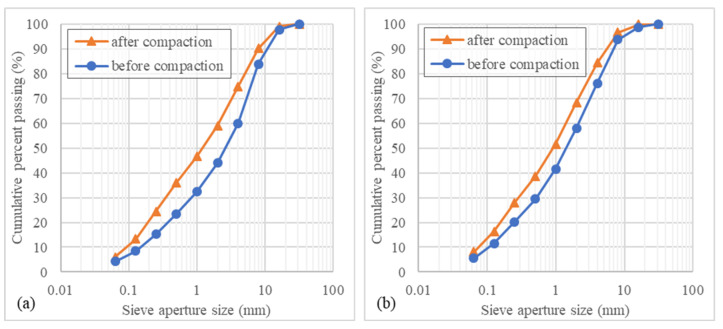
Particle size distribution of red scoria (**a**) and black scoria (**b**) before and after compaction.

To evaluate the effect of the compaction energy used to produce the specimens, the particle size distribution was identified before and after the compaction process (Figure 5). Following the compaction process, the specimens were manually disaggregated, and a new analysis of particle size distribution was conducted. For both scoria types, the compaction energy affected the particle size distribution, highlighting the low shock resistance of the coarse particles of the scoria. Regarding the particle size distributions presented in Figure 5, before compaction, the fineness modulus was 5.3 and 4.7, and after compaction was 4.5 and 4.1 for the red scoria and black scoria, respectively. A lower fineness modulus is associated with a finer aggregate. The relative reduction of the fineness modulus was similar for both scoria types (−15% for red scoria and −12% for black scoria).

### 3.2. Mechanical Performance

#### 3.2.1. Indirect Tensile Strength

**Figure 6:**
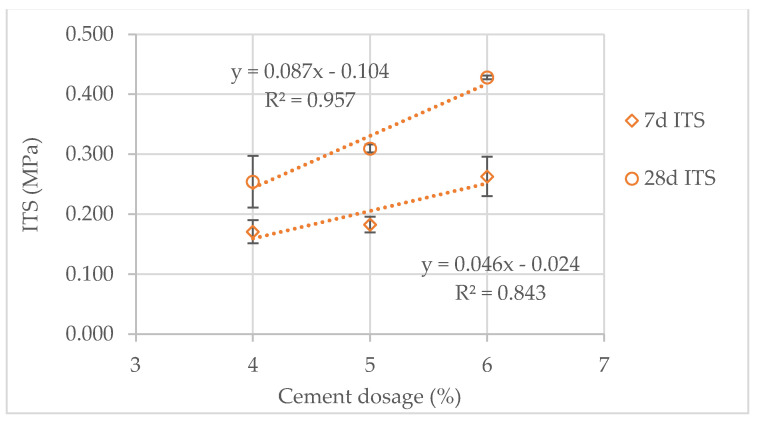
ITS of the red CTS after 7 and 28 days of curing.

**Figure 7:**
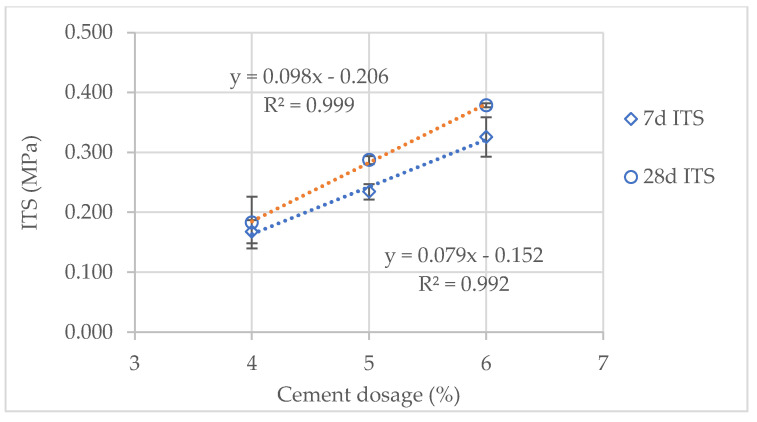
ITS of the black CTS after 7 and 28 days of curing.

As expected, the indirect tensile strength (ITS) of the cement-treated scoria (CTS) specimens increased with cement dosage and curing age (Figure 6 and Figure 7). After seven days of curing, with 4% cement dosage, both CTS types presented similar ITS. However, with 5% and 6% cement dosage, the black CTS showed 26% higher ITS compared with the red CTS. After 28 days of curing, the red CTS showed the highest ITS (15% on average) for all cement dosages. Thus, the increase in ITS with curing age was higher for the red CTS than for the black CTS. For the red CTS, the increasing ITS from the 7th to 28th curing day were 49%, 70% and 63%, for the cement dosages of 4%, 5% and 6%, respectively. Figures for the same circumstances for the black CTS were 9%, 23% and 16%, for the cement dosages of 4%, 5% and 6%, respectively. Regarding the increase in cement dosage, both CTS types exhibited an approximately linear increase in ITS (Figure 6 and Figure 7). Thus, for the red CTS, ITS’ increasing at 7 days and 28 days of curing was, on average, 46 kPa and 87 kPa by each 1% increase in cement dosage. Figures for the same circumstances for the black CTS were 79 kPa and 98 kPa. 

Generally, the ITS results indicate adequate behavior of the CTS, within the expectations of having cement-treated materials. However, the red CTS seemed more sensitive to curing age, whereas the black CTS seemed more sensitive to cement dosage. These minor variations can be partially explained by the differences within the materials and test method repeatability. Regarding infrastructure agency specifications, the South African Pavement Engineering Manual, SAPEM [31] indicates minimum values for the 7-day ITS of cementitiously stabilized materials (CSM). The red CTS with 6% cement dosage qualifies for the class CSM-C4 that requires a minimum 7-day ITS of 0.2 MPa. The red CTS with 5% cement dosage has ITS values very close to this limit. Therefore, the black CTS with 5% or 6% cement dosages qualifies for the class CSM-C4.

#### 3.2.2. Unconfined Compressive Strength

**Figure 8:**
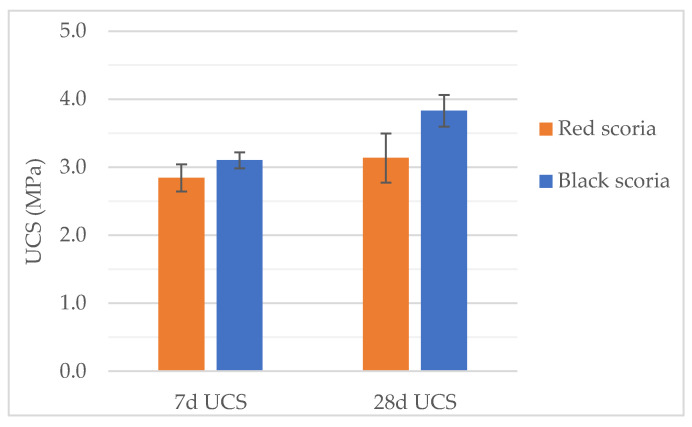
UCS of the red CTS and black CTS after 7 and 28 days of curing.

The unconfined compressive strength (UCS) tests were conducted in specimens with 5% of cement dosage for both CTSs’. In the UCS tests, the black CTS showed the highest strength at both 7-days and 28-days curing ages (Figure 8). Compared with the red CTS, the black CTS presented higher UCS by 9% and 22%, at 7 and 28 days of curing, respectively. Regarding the evolution of UCS with curing age, between the 7th and the 28th day of curing, the red CTS presented an increase of 10% while the black CTS increased by 23%. In this case, the evolution of UCS with curing age for the red CTS seems comparatively low. However, the higher variation presented by the red CTS specimens can explain such results. The coefficient of variation of the UCS results for the red CTS was 7% and 12%, while for the black CTS was 4% and 6%, at 7-days and 28-days of curing, respectively. Furthermore, the variations in UCS can be partially explained by the repeatability of the test method and small differences within the properties of the materials such as particle size distribution and particle density.

Regarding the relationship between ITS and UCS, the ratio ITS/UCS can generally vary from 1:7 to 1:15 [31]. In the case of the red CTS, the ratio ITS/UCS was 15.6 and 10.1, at the 7-day and 28-day curing age, respectively. In the case of the black CTS, the ratio ITS/UCS was 13.2 and 13.3, at the 7-day and 28-day curing age, respectively. Thus, both CTS materials presented a behavior similar to traditional cement-treated materials. 

Both CTS materials with 5% cement comply with the criteria for cement-bound granular mixtures indicated by several infrastructure agencies. For instance, the Portuguese Infrastructure agency [30] requires a minimum 28-day UCS of 1 MPa. The Australian Guide for Pavement Technology [32] indicates that cemented-treated material should have a minimum 28-day UCS of 2 MPa. Regarding the SAPEM specifications [31], both CTS qualify for the class CSM-C3 which requires a minimum 7-day UCS of 1.5 MPa, as well as for the class CSM-C4 (matching with the classification obtained according to the ITS criteria) which requires a minimum 7-day UCS of 0.75 MPa. 

## 4. Conclusions

This study investigated cement-treated volcanic scoria as a paving material for low-traffic rural roads. The geotechnical properties of two types of volcanic scoria were characterized, and the mechanical performance of the respective cement-treated mixture was obtained. The geotechnical properties of the volcanic scoria were characterized through particle size distribution, particle density, water absorption, sand equivalent, methylene blue, organic matter and modified Proctor compaction tests. The mechanical performance of the cement-treated scoria was evaluated through unconfined compressive strength and indirect tensile strength tests.

The geotechnical characterization highlighted the adequate properties of both scoria types, showing some differences between them, as expected for this type of natural volcanic material.

Regarding unconfined compressive strength and indirect tensile strength, both scoria types presented a behavior within the expectations of cement-treated materials, exhibiting an intermediate performance between typical soil-cement and good quality cement-treated aggregate materials. 

The volcanic scoria treated with moderate cement content (4% to 6%) presented good mechanical performance, which indicates that it is suitable for application in cement-bound granular layers as a subbase or base layer for main roads and constituting the pavement for low-traffic rural roads. Furthermore, the use of cement-treated scoria is a cost-effective alternative to the current status in terms of Azorean pavement technology, with the advantages of using locally available materials and more straightforward construction equipment that is more readily available.

Further research must be conducted to evaluate with more detail the behavior of the cement-treated scoria when under more traffic-like loading conditions, for instance, flexural strength, flexural elastic modulus and fatigue. In addition, further insights regarding long-term properties must be obtained, possibly through durability tests, for example, by the wetting-and-drying method.

## Figures and Tables

**Table 1:** Test methods used for the geotechnical characterization of scoria.

Test/Parameter	Test Standard
Particle size distribution	EN 933-1 [18]
Particle density and water absorption	EN 1097-6[19]
Fineness modulus	ASTM C125[20]
Sand equivalent	EN 933-8[21]
Methylene blue	EN 933-9[22]
Organic matter	ASTM D2974[23]

**Table 2:** Properties of the red and black scoria.

Parameter		Type of Scoria	
		Red	Black
Fraction 0.063/4—Apparent particle density	Mg/m^3^	2.37	2.48
Fraction 0.063/4—Particle density on an oven-dried basis	Mg/m^3^	2.29	2.45
Fraction 0.063/4—Particle density on a saturated and surface-dried basis	Mg/m^3^	2.32	2.46
Fraction 0.063/4—Water absorption	%	1.53	0.47
Fraction 4/16—Apparent particle density	Mg/m^3^	2.13	2.01
Fraction 4/16—Particle density on an oven-dried basis	Mg/m^3^	1.63	1.42
Fraction 4/16—Particle density on a saturated and surface-dried basis	Mg/m^3^	1.87	1.72
Fraction 4/16—Water absorption	%	14.35	20.47
Sand equivalent	%	89.2	81.4
Methylene blue	g/kg	1.25	0.22
Organic matter	%	0.65	0.09
